# Immunomodulatory Effects of the Nutraceutical Garlic Derivative Allicin in the Progression of Diabetic Nephropathy

**DOI:** 10.3390/ijms19103107

**Published:** 2018-10-11

**Authors:** Abraham Said Arellano Buendía, Montserrat Tostado González, Omegar Sánchez Reyes, Fernando Enrique García Arroyo, Raúl Argüello García, Edilia Tapia, Laura Gabriela Sánchez Lozada, Horacio Osorio Alonso

**Affiliations:** 1 Renal Physiopathology Laboratory, Department of Nephrology, Instituto Nacional de Cardiología “Ignacio Chávez” México City 14080, Mexico; neoabraham_7@hotmail.com (A.S.A.B.); pochi859@hotmail.com (M.T.G.); tuzome_sanrey23@hotmail.com (O.S.R.); jonibertojr@hotmail.com (F.E.G.A.); ediliatapia@hotmail.com (E.T.); lgsanchezlozada@gmail.com (L.G.S.L.); 2 Departamento de Genética y Biología Molecular, Centro de Investigación y de Estudios Avanzados-IPN, México City 07360, Mexico; raularguellogarcia@yahoo.com

**Keywords:** allicin, diabetic nephropathy, inflammation, oxidative stress

## Abstract

Diabetic nephropathy (DN) is presently the primary cause of chronic kidney disease and end-stage renal disease (ESRD). It has been suggested that inflammation and oxidative stress, in addition to or in concert with the metabolic changes, plays an important role in the maintenance and progression of the disease. Therefore, attenuating or blocking these mechanisms may be a therapeutic target to delay the progression of the disease. Diallyl thiosulfinate (allicin), a compound derived from garlic, inhibits free radical formation, increases glutathione synthesis and decreases the levels of proinflammatory molecules in vitro. This research aimed to assess the effect of allicin on oxidative stress and inflammation-induced diabetes. Animals were divided into control and diabetes (streptozotocin 50 mg/kg i.p.), and maintained for 30 days. After 30 days, the group of diabetic animals was subdivided into diabetes and allicin-treated diabetes (16 mg/kg/day oral gavage). The three experimental groups were maintained for another month. We analyzed the status of renal function, oxidative stress and proinflammatory cytokines. The untreated diabetic group showed hyperglycemia and increased diuresis, creatinine clearance, proteinuria, glycosuria and urinary excretion of *N*-acetyl-β-d-glucosaminidase (NAG), as well as increased oxidative stress and the expression of interleukin 1β (IL-1β), IL-6, nuclear factor kappa beta (NFκβ) and transforming growth factor-β1 (TGF-β1) in plasma and kidney. In contrast, the inhibitor of NFκβ (Iκβ) is decreased in the cortex. It has been demonstrated that the allicin treatment decreases hyperglycemia, polyuria, and NAG excretion. The oxidative stress and proinflammatory cytokines were also reduced by the allicin treatment. In conclusion, allicin delays the progression of diabetic nephropathy through antioxidant and anti-inflammatory mechanisms.

## 1. Introduction

Diabetic nephropathy (DN) is an irreversible progressive disorder and a long-standing complication of diabetes. DN accounts for 12–55% of cases of end-stage renal diseases (ESRD) [1]. Different pathophysiological mechanisms contribute to the development and progression of DN. The mechanisms include hemodynamic changes such as hyperfiltration, synthesis of growth factors, cytokines and release of reactive oxygen species (ROS) [2]. Using an experimental model of diabetes, we previously described increased expression of the proinflammatory interleukins 1β (IL-1β) and interleukin 6 (IL-6), of which the presence is associated with renal dysfunction, as well as an increase in oxidative stress, in mesangial matrix index and fibrosis [3]. Other studies have reported that the development of DN is associated with infiltration of inflammatory cells, increase in plasma levels of C-reactive protein (CRP) and proinflammatory cytokines such as vascular cell adhesion molecule-1 (VCAM-1), interleukins (IL-1, IL-6, and IL-18) and tumor necrosis factor-α (TNF-α) [4]. On the other hand, oxidative stress and overgeneration of reactive species stimulate systemic chronic kidney inflammation, leading to a vicious circle empowering the progression of DN [5,6].

The evidence of inflammatory processes involved in the progression of DN is strongly supported in the scientific literature [7]. Based on the above-described evidence on the role of inflammation in progression of DN, the pharmacological approaches to treating diabetes should not only correct hyperglycemia, but also target chronic inflammation to prevent the development of metabolic and microcardiovascular complications.

Diallyl thiosulfinate (allicin) is a major component formed when garlic (*Allium sativum*) is crushed or ground [8]. Allicin is considered as a panacea as it has a broad spectrum of physiological activities such as antihypertensives [9,10], antioxidants [9], antidiabetics [11], cardioprotectives [10], nephroprotectives [11], and immunomodulators [12,13]. As far as nephroprotective mechanisms of allicin in experimental DN are concerned, recent studies have shown that this garlic derivative partially reverses the increased expression of transforming growth factor β (TGF-β) and the activation (i.e., phosphorylation) of its downstream target extracellular signal-regulated kinase ½ (ERK1/2) that drive renal epithelial-mesenchymal transition and fibrosis [11].

On the other hand, in spite of the aforementioned importance of inflammatory processes in DN as well as the upstream modulation of TGF-β signaling by ROS [14], the likely anti-inflammatory and antioxidant effects of allicin and their underlying mechanisms in the progression of DN remain unclear.

In this work, we hypothesize that allicin attenuates the inflammation and oxidative stress-induced diabetes and eventually delays the progression of DN, and experimentally investigate the effect of allicin on proinflammatory cytokines in diabetes using streptozotocin-treated rats.

## 2. Results

### 2.1. Effects of Allicin on Blood Glucose, Body Weight, Diuresis, Serum Creatinine, Creatinine Clearance, Proteinuria, Glycosuria and Urinary Excretion of N-acetyl-β-d-glucosaminidase

**Figure 1:**
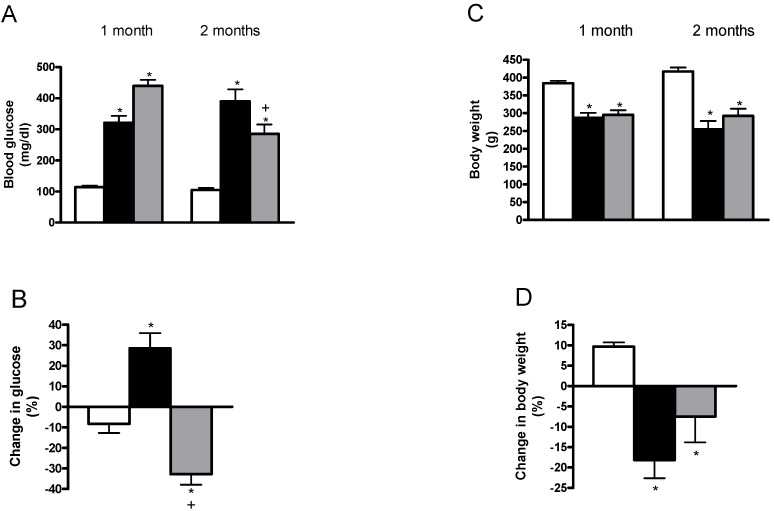
Comparisons of blood glucose (**A**,**B**) and body weight (**C**,**D**) for the control, untreated diabetic and allicin-treated diabetic groups. White bars, black bars and grey bars indicate the control, untreated diabetic and allicin-treated diabetic groups, respectively. Data represent the average of values from all the animals (*n* = 6) in a given group ± standard error of the mean (SEM) (* *p* < 0.05 vs. the control group; ^+^ *p* < 0.05 vs. the diabetic groups).

At the beginning of treatments (the first month), both groups of untreated diabetic and allicin-treated diabetic rats had higher blood glucose levels as compared with the control group (Figure 1A). Moreover, the hyperglycemia in the allicin-treated diabetic group was more elevated, as compared with the untreated diabetic group (Figure 1A).

At the end of the treatments (the second month), the blood glucose levels were higher in untreated and allicin-treated diabetic rats, compared with the control group (Figure 1A). In the allicin-treated diabetic rats, the hyperglycemia was lower than that of untreated diabetic rats (Figure 1A). To clarify the effects of allicin on hyperglycemia, we calculated the percentage change in blood glucose between the first and the second months. The results showed that allicin treatment markedly decreased the blood glucose values as compared to the untreated diabetic rats (Figure 1B), which showed a significantly higher percentage of change in hyperglycemia compared to the control group (Figure 1B).

Throughout the experiment, the control group steadily gained weight, whereas the diabetic rats failed to gain body weight in comparison to the control group, which was as expected (Figure 1C). The allicin treatment on diabetic rats did not cause statistical differences as compared with the untreated diabetic group. To analyze in detail the gain or loss in body weight, we calculated the percentage change in body weight between the first and the second months. As expected, the control group gained body weight and the two diabetic groups lost weight (Figure 1D). It was also shown that allicin treatment prevented the loss in body weight as compared with the untreated group (Figure 1D).

**Figure 2:**
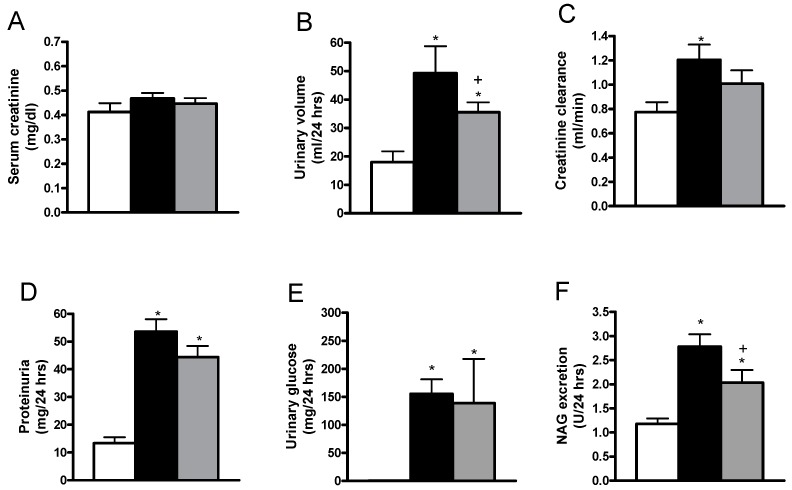
Comparisons of (**A**) serum creatinine, (**B**) urinary volume, (**C**) creatinine clearance, (**D**) proteinuria, (**E**) urinary glucose, and (**F**) urinary excretion of *N*-acetyl-β-d-glucosaminidase (NAG) for the control, untreated diabetic and allicin-treated diabetic groups. White bars, black bars and grey bars indicate the control, untreated diabetic and allicin-treated diabetic groups, respectively. Data represent the average of values from all the animals (*n* = 6) in a given group ± SEM (* *p* < 0.05 vs. the control group; ^+^ *p* < 0.05 vs. the diabetic groups).

Additionally, we analyzed the diuresis, serum creatinine, creatinine clearance, proteinuria, glycosuria and the urinary excretion of *N*-acetyl-β-d-glucosaminidase (NAG). We did not find changes in serum creatinine in the two diabetic groups compared with the control group. In the diabetic rats, the diuresis, creatinine clearance, proteinuria, urinary glucose and the urinary excretion of glucose and NAG were increased as compared with the control group (Figure 2A–F), which is a well-known feature of diabetes-induced renal dysfunction. These data showed the typical spectrum of the disease, hyperglycemia, decrease in body weight, and increase in diuresis and glycosuria. It can be seen that the allicin treatment induced a decrease in urinary volume, and urinary excretion of NAG (Figure 2A,B,F) and improved the creatinine clearance and proteinuria (Figure 2C,D). In contrast, the glycosuria of the diabetic rats treated with allicin was similar to that of the untreated diabetic rats (Figure 2E).

### 2.2. Effects of Allicin on Oxidative Stress Markers

**Figure 3:**
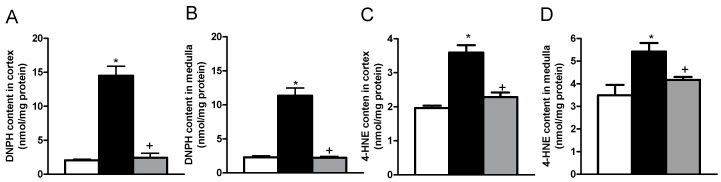
Effects of allicin on oxidative stress markers in kidney; protein oxidation in (**A**) cortex and (**B**) medulla, and lipid oxidation in (**C**) cortex and (**D**) medulla. White bars, black bars and grey bars indicate the control, untreated diabetic and allicin-treated diabetic groups, respectively. Data represent the average of values from all the animals (*n* = 6) in a given group ± SEM (* *p* < 0.05 vs. the control group; ^+^ *p* < 0.05 vs. the diabetic groups).

We determined the protein oxidation and the lipid oxidation as indicators of oxidative stress in kidney. In the kidney cortex of the untreated diabetic group, the contents of 2,4-dinitrophenylhydrazine (DNPH) as an indicator of protein oxidation were higher in cortex and medulla as compared with the content of DNPH in the kidney of the control group (Figure 3A,B). Moreover, the contents of 4-Hydroxynonenal (4-HNE) in the cortex and medulla were higher in the untreated and allicin-treated diabetic groups than those with the control group (Figure 3C,D). Thus, the results confirmed an increase in oxidative stress in diabetes [3]. It was also found that the allicin treatment decreased the oxidative stress, as evidenced by lower contents of DNPH and 4-HNE in the allicin-treated diabetic group, as compared with the untreated diabetic animals.

### 2.3. Effects of Allicin on Proinflammatory Cytokines

**Figure 4:**
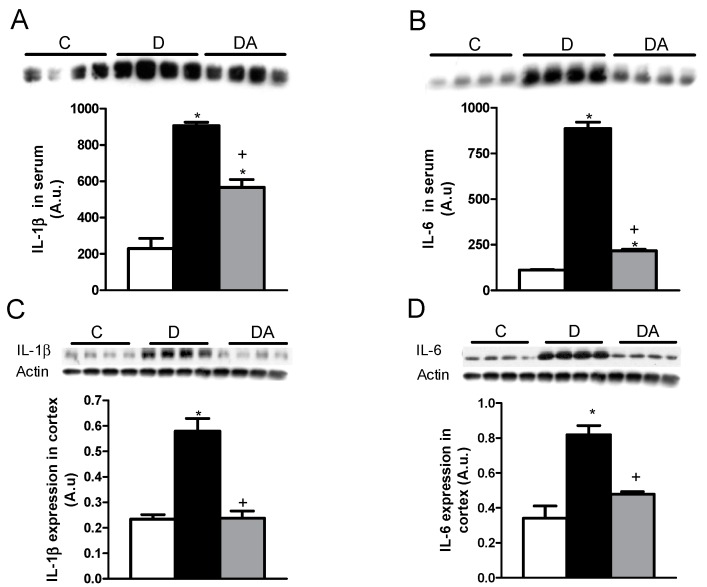
Effects of allicin on proinflammatory cytokines in serum with expressions of interleukin 1 beta (IL-1β) (**A**) and interleukin 6 (IL-6) (**B**) and in cortex with expressions of IL-1β (**C**) and IL-6 (**D**). C, D and DA in figures represent the control, untreated diabetic and allicin-treated diabetic groups, respectively. Data represent the average of values from all the animals (*n* = 6) in a given group ± SEM (* *p* < 0.05 vs. the control group; ^+^ *p* < 0.05 vs. the diabetic groups).

To assess the effect of allicin on the levels of proinflammatory cytokines, we analyzed the expression of IL-1β and IL-6 in plasma and kidney using rats with one month of diabetes evolution and another month under treatment with allicin. In these analyses, the untreated diabetic rats showed an increase in IL-1β and IL-6 in plasma in comparison with the control group (Figure 4B). The allicin treatment decreased IL-1β as well as IL-6 in plasma as compared with the untreated diabetic rats (Figure 4A,B). The anti-inflammatory effect of allicin was better observed at the level of IL-6 down-regulation levels than at the IL-1β level (Figure 4A,B).

In the kidney cortex, the expressions of IL-1β and IL-6 were increased in diabetic rats when compared with the control group (Figure 4C,D). The allicin treatment prevented the elevation of these proinflammatory cytokines as compared with the diabetic animals untreated (Figure 4C,D). These data demonstrated an anti-inflammatory effect of allicin at systemic and renal levels (Figure 4).

**Figure 5:**
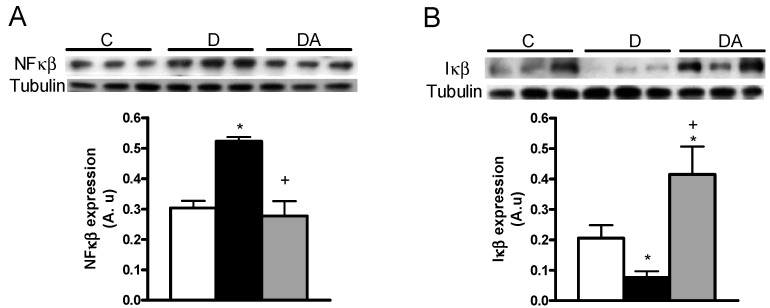
Effects of allicin on the expression of nuclear factor kappa beta (NFκβ) (**A**) and its inhibitor (Iκβ) (**B**) in the kidney cortex. C, D and DA in figures represent the control, untreated diabetic and allicin-treated diabetic groups, respectively. Data represent the average of values from all the animals (*n* = 6) in a given group ± SEM (* *p* < 0.05 vs. the control group; ^+^ *p* < 0.05 vs. the diabetic groups).

It has been suggested that nuclear factor-κβ (NFκβ) is associated with inflammatory disease; we therefore analyzed the protein expression of NFκβ and its inhibitor (Iκβ). In the kidney cortex of the untreated diabetic group, the expression of NFκβ was increased as compared with the control group (Figure 5A). It can be seen that the allicin treatment in diabetic rats prevented the increase in the NFκβ expression in the kidney cortex (Figure 5A).

Conversely, the expression of the Iκβ was lower in the diabetic group compared with the control group (Figure 5B) and the allicin treatment prevented the decrease of Iκβ when compared with the untreated diabetic group (Figure 5B). In addition, allicin upregulated the Iκβ expression as compared with both the untreated diabetic and the control groups (Figure 5).

**Figure 6:**
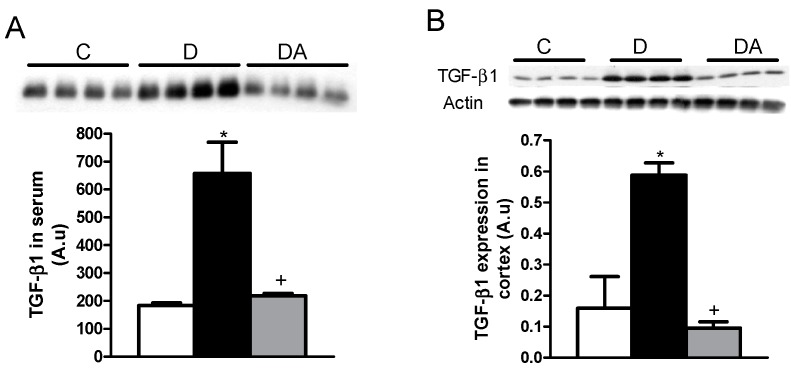
Effects of allicin on transforming growth factor beta (TGF-β1) in plasma (**A**) and kidney cortex (**B**). C, D and DA in figures represent the control, untreated diabetic and allicin-treated diabetic groups, respectively. Data represent the average of all the animals (*n* = 6) in a given group ± SEM (* *p* < 0.05 vs. the control group; ^+^ *p* < 0.05 vs. the diabetic groups).

To elucidate the anti-fibrotic effects of allicin, the transforming growth factor beta (TGF-β1) protein was analyzed. As shown in Figure 6A,B, TGF-β1 expression in the serum and kidney cortex from diabetic rats was significantly increased. Meanwhile, the allicin treatment markedly decreased the TGF-β1 expression in the serum and the renal cortex (Figure 6A,B).

## 3. Discussion

DN is a major cause of death from diabetes in the world [1]. It is well known that the major causative agent of kidney disease is chronic hyperglycemia. However, recent studies showed that oxidative stress and inflammation, in addition to hyperglycemia, significantly contribute to the progression of this microvascular complication of diabetes [3,4,5,6,7]. Therefore, an adequate control of blood glucose levels, in addition to careful management of the nontraditional factors in diabetes, is necessary to delay the progression of DN. On the other hand, due to the disease′s complexity and the limited availability of drugs, it is necessary to search alternative therapies to delay or prevent the disease developing to terminal stages. In the present study, allicin was selected for evaluation of its probable beneficial effects on renal function as well as immunomodulatory effects in rats with STZ-induced diabetes.

The experimental results showed that the induction of diabetes provoked a stable increase in the blood glucose level in STZ-treated animals with a concomitant decrease in body weight. Additionally, the diabetic group showed an increase in diuresis, creatinine clearance, proteinuria, glycosuria and urinary excretion of NAG, which are considered as hallmarks of renal dysfunction in diabetes. These results are in good agreement with other studies, which reported proteinuria or albuminuria in this experimental model of diabetes [3,11,12,15]. It has been demonstrated that one month of allicin treatment prevented the loss of body weight in diabetic animals. Moreover, the blood glucose levels in this allicin-treated group were lower when compared with those values at the beginning of treatment. In addition, the allicin treatment started and included the diabetic rats with the highest values of hyperglycemia (440 ± 20 mg/dL vs. 322 ± 20 for the allicin-treated diabetic and untreated diabetic, respectively). Other studies reported that the allicin treatment decreases the blood glucose levels and prevents the loss of body weight in diabetic animals [11,12,16]; however, the exact mechanisms for such effects are unknown. On the other hand, it has been reported that *S*-allyl cysteine sulfoxide (alliin) [17], the precursor of allicin, maintains glucose homeostasis, improves insulin sensitivity [18], decreases fasting glucose levels and increases insulin levels in diabetic rats. Moreover, the effects of alliin have a similar effect to those of glibenclamide, glyclazide or insulin [19,20].

Hyperglycemia during diabetes provokes elevation of diuresis, proteinuria, glycosuria and excretion of NAG, which are considered as significant indicators of renal dysfunction in diabetes [3,15]. The results in the present study indicated that experimental diabetes led to renal dysfunction. Meanwhile, the allicin treatment decreased glycaemic levels, hence diuresis, creatinine clearance and urinary excretion of NAG, which validates the main renal benefits of this garlic compound and concur with other previous studies using allicin in this model [11].

Regarding the mechanism underlying the effects of allicin in renal function during DN, chronic hyperglycemia through activation of various signaling pathways leads to the increased formation of free radicals and ROS. It is well known that oxidative stress plays an important role in the development and progression of DN by targeting a plethora of cellular proteins and lipids [21]. We used 4-HNE and DNPH as indicators of the end-product of lipid peroxidation and protein carbonylation to estimate the oxidative stress in diabetic kidney. Here, our data showed increased levels of 4HNE and DNPH in the cortex and the renal medulla of the diabetic group. The oxidative stress in diabetes was attenuated at protein and lipid levels by the allicin treatment. Previously, using an experimental model of chronic kidney disease (CKD) by renal ablation we observed an antioxidant effect of allicin by a mechanism dependent on the nuclear factor erythroid 2-related factor 2/Kelch ECH associating protein 1 (Nrf2/Keap1) pathway [9]. Other studies reported antioxidant properties of allicin by scavenging free radicals and hydroxyl radicals (OH•) [22] and indirectly up-regulating the phase II detoxifying enzymes [23].

Recent studies have shown evidence supporting the hypothesis that inflammation also plays a key role in the development and progression of DN [2,3,7]. Hyperglycemia stimulates resident and non-resident renal cells to produce cytokines and growth factors that contribute to the development of renal injury [7]. In the present study, we assessed the effect of allicin on proinflammatory cytokines. Our results showed that chronic hyperglycemia increased IL-1β and IL-6 in serum and kidney cortex. The allicin treatment decreased both proinflammatory cytokines and these effects were exerted at the systemic and tissue levels. In vitro and in vivo allicin modulates the production of IL-1β, IL-6 and TNF-α at both the mRNA and protein levels [24,25,26]. Other studies described that allicin has anti-inflammatory effects [27] and suppresses both oxidative stress and proinflammatory cytokines [IL-1, TNF-α and Monocyte Chemoattractant Protein-1 (MCP-1)] [26,28].

One of the most important transcription factors in the pathogenesis of DN is NFκβ, a ubiquitous transcription factor that is activated by a wide variety of stimuli present in diabetic milieu, such as cytokines, oxygen radicals and mechanical forces [29]. In the present study, the expression of NFκβ was increased in the kidney cortex from diabetic rats. In contrast, the expression of Iκβ, the repressor of NFκβ, was decreased. Moreover, the allicin treatment suppressed these alterations induced by the diabetes onset. In fact, the expression of Iκβ was higher in the allicin-treated group than that in the control group. To the best of our knowledge, this is the first study that reports the effects of allicin on NFκβ and its repressor on the progression of DN.

It has been suggested that the anti-inflammatory mechanism of allicin is dependent on the inhibition of NFκβ activity, which is responsible for the transcription of proinflammatory cytokines [29]. In addition, allicin significantly reduces the expression of Iκβ, the regulatory unit of NFκβ, which is essential for the activation of transcription of proinflammatory cytokines [29].

On the other hand, allicin reduces the activation of NFκβ, the nuclear translocation and the phosphorylation of Iκβ induced by IL-1 [25]. Moreover, allicin suppresses IL-1β-induced phosphatidylinositol 3-kinase/protein kinase B (PI3K/AKT) phosphorylation, a pathway involved in the regulation of NFκβ [25]. In fructose-fed diabetic rats, the administration of garlic homogenate decreases cardiac hypertrophy, NFκβ activity and oxidative stress through the PI3K/AKT/Nrf2-Keap1-dependent pathway [30]. The authors noted that the homogenate of garlic is rich in allicin and other compounds such as γ-glutamyl-*S*-allyl-l-cysteine, alliin, *S*-Allyl-l-cysteine, deoxyalliin and vinyldithiin [30].

The beneficial effects of allicin are also reflected by TGF-β levels, which are increased in the diabetic group and normalized by the allicin treatment. In HK-2 cells, high glucose concentration induces the expressions of TGF-β1 and p-ERK1/2 in a dose-dependent manner, and allicin inhibits this signaling pathway, which is associated with epithelial-myofibroblast transdifferentiation [31]. Allicin has shown an antifibrotic effect in the myocardial infarction by down-regulation of the TGF-β/Smads pathway [32].

**Figure 7:**
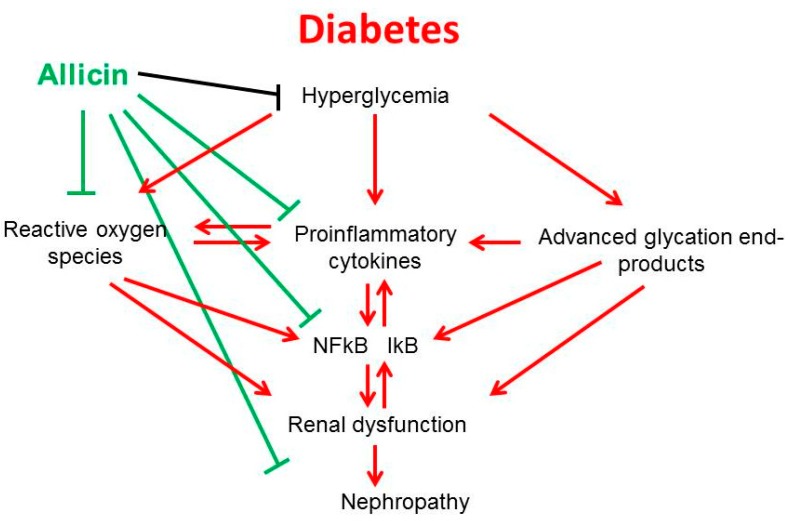
Anti-inflammatory mechanism of allicin in diabetic kidney. Red lines show the effect of diabetes and green lines effect of allicin.

As mentioned, the diabetic milieu is a complex environment where the hyperglycemia plays a key role in the activation of several metabolic pathways. The hyperglycemia stimulates the free radical production. On the other hand, experimental evidence shows that the hyperglycemia up-regulate the synthesis of proinflammatory cytokines. Moreover, glucose autoxidation leads to the formation of advanced glycation end products (AGEs). The ROS and AGEs can enhance the synthesis of proinflammatory cytokines empowering the inflammation in diabetes. At the same time, these mechanisms result in the activation of NFκβ, which also, down the line, activates the gene transcription of proinflammatory and profibrotic cytokines that leads to the impairment of renal function (Figure 7).

The relationship between oxidative stress and inflammation in diabetes plays a key role in the progression of DN, so blocking these pathological events can be useful in the treatment of the disease. Nevertheless, by virtue of the complexity of the disease, it seems unlikely that one single therapy may be sufficient to arrest the progression of pathology. In the present study, the beneficial effects from allicin on DN were shown, including attenuation of hyperglycemia, oxidative stress and inhibition of inflammation through the down-regulation of NFκβ/Iκβ and its target genes IL-1 and IL-6. Thus, in the search for new therapeutic strategies that assist in slowing down the progression of DN towards advanced chronic kidney disease, allicin is a potential candidate, which offers diverse benefits including the molecular events that still remain unknown. Other studies have reported beneficial effects of allicin on glucose, immune response, antioxidant and fibrosis, but they administered allicin simultaneously to the injury or before the damage occurs. Thus, the beneficial effects of allicin are preventive. That situation in real life does not happen, as the therapy starts in a patient when the pathology is well established and advanced. For that reason, we designed the present study, allowing diabetes to install for one month before the administration of allicin as a therapy; this manipulation was with the aim of extrapolating our results to diabetic patients, who often do not know their disease.

In conclusion, our study showed evidence that allicin was effective in inhibiting oxidative stress, systemic and renal inflammation in established diabetes. Additionally, we observed anti-inflammatory effects presumably mediated directly or indirectly by their effects on NFκβ. In addition, the presented study is the first one that explored the anti-inflammatory effect of allicin using an experimental model of DN with inflammation and oxidative stress established [3]. Thus, our results have potential application in further studies on clinical medicine, because the treatments should target to block or delay the progression of the disease, when the disease is advanced and more than one pathogenic mechanism is clearly established.

## 4. Methods

### 4.1. Reagents

STZ, dichloromethane, diallyl disulfide, hydrogen peroxide, 4-nitrophenol, 4-nitrophenyl-*N*-acetyl-β-d-glucosaminide, tetramethoxypropane, 2,4-dinitrophenylhydrazine, methanesulfonic acid, 1-methyl-2-phenylindole were purchased from Sigma-Aldrich (St. Louis, MO, USA). All other chemicals used were of the highest purity available. IL-1β antibody: sc-1250; IL-6 antibody: sc-57315; TGF-β1 antibody: sc-130348 and Actin antibody: sc-8432 were purchased from Santa Cruz Biotechnology (Dallas, TX, USA). Anti-NFkβ p65 GTX102090 and Ikβ beta GTX82797 were from GeneTex (Irvine, CA, USA). Goat anti-rabbit and anti-mouse IgG-HRP were purchased from Cell Signaling Technology (Danvers, MA, USA).

### 4.2. Experimental Design

**Figure 8:**
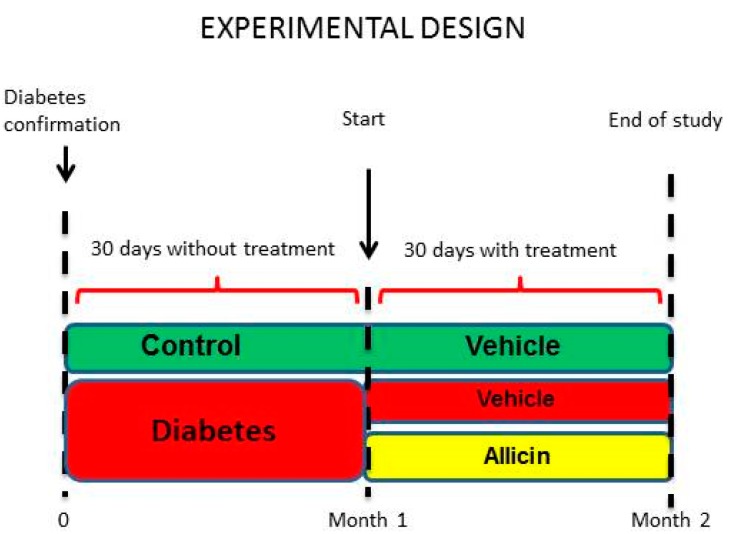
Schematics of experimental design. To examine the effects of allicin on inflammation, we used an experimental model of diabetes. For diabetes induction, streptozotocin (50 mg/kg) was administered intraperitoneally to Wistar male rats. After diabetes confirmation, the rats were maintained for 1 month with food and water *ad libitum*. One month after diabetes confirmation, the blood glucose levels were measured, and the diabetic rats were subdivided into diabetic or allicin-treated diabetic groups. Once the three experimental groups were formed (control, diabetic and allicin-treated diabetic), the administration of the treatments started daily for 1 month.

Male Wistar rats weighing 280–310 g were used. Animals were randomly divided into two groups: control (C, *n* = 6) and diabetic (D, *n* = 14). Diabetes was induced by a single administration of STZ (50 mg/kg i.p.) dissolved in citrate buffer (0.1 M, pH 4.5). The control group received the same volume of citrate buffer. After 72 h of STZ administration, blood glucose concentration was determined (Accu-Chek sensor comfort, Roche Diagnostics, Basel, Switzerland). Ninety percent of rats reached a serum glucose level over 250.0 mg/dL and those animals were considered diabetic for further studies (*n* = 12). C and D rats were maintained for 30 days on a laboratory diet with water *ad libitum* (Figure 8).

After 30 days of follow-up, the diabetic rats were further divided into two groups. The first group of diabetic animals with blood glucose values of approximately 321.5 ± 19.5 mg/dL was administered only vehicle; this group was considered as diabetic control or untreated. The second group of diabetic animals with pointedly selected blood glucose values of approximately 440 ± 19.5 mg/dL, was assigned to be treated with allicin (16 mg/kg/day gavage) for one month (Figure 8). At the end of the experiment (two months) and after completion of the experimental protocols, blood and urine samples were collected. Additionally, the rats were euthanized and the kidney was obtained, separated in the cortex and medulla, snap frozen in liquid nitrogen and stored at −70 °C until further analysis.

### 4.3. Ethics Statement

This study was performed in accordance with the Guide for the Care and Use of Laboratory Animals, published by the U.S. National Institutes of Health, and approved by the Research Committee of the National Institute of Cardiology Ignacio Chávez and by the Mexican Federal Regulation for animal experimentation and care (NOM-062-ZOO-2001) and for the disposal of biological residues (NOM-087-ECOL-1995).

### 4.4. Allicin

Allicin was produced by oxidation of diallyl disulfide as previously reported [9]. For stabilization and storage, allicin was resuspended in water at 2.5% (*w*/*v*) and kept at −70 °C until used.

### 4.5. Renal Function

At the end of the experiment, the rats were placed in metabolic cages (Nalgene, Rochester, NY, USA) to collect 24-h urine. Urine samples were centrifuged at 5000× *g* for 15 min to remove debris, and the supernatant was analyzed. The variables measured were diuresis, creatinine (Creatinine Assay Kit (ABCAM, Cambridge, MA, USA)), glucosuria (IL 300 plus, Clinical Chemistry Analyzer, Instrumentation Laboratory, Bedford, MA, USA), and the urinary NAG activity (spectrophotometric assay) in urine sample as markers of tubular injury.

#### Measurement of *N*-acetyl-β-d-glucosaminidase (NAG) Activity

For the determination of NAG activity in urine samples, 4-nitrophenyl-*N*-acetyl-β-d-glucosaminide was used as the substrate. One unit of enzymatic activity (U) represents the amount of enzyme that hydrolyses 1 μmol of substrate per min at 37 °C. The results were expressed as units/day (U/24 h).

### 4.6. Evaluation of Renal Oxidative Stress

#### 4.6.1. Determination of Lipid Peroxidation

4-HNE was measured using a standard curve of tetramethoxypropane. A mixed solution of 1-methyl-2-phenylindole and acetonitrile:methanol (volume ratio, 3:1) was added to the kidney cortex or medulla homogenates and the reaction was started with 37% HCl or methanesulfonic acid plus FeCl_3_, to measure 4-HNE separately. Optical density was measured at 586 nm after 1 h of incubation at 45 °C. Data were expressed as nmol of 4-HNE per milligram of protein (nmol HNE/mg protein).

#### 4.6.2. Measurement of Oxidized Proteins

The presence of carbonyl groups in the proteins was measured using the reaction with DNPH. Protein carbonyl groups were estimated by using the molar absorption coefficient of 22,000 M^−1^∙cm^−1^ for DNPH derivatives, and its concentration was expressed as nmol carbonyl groups/mg protein. The guanidine solution was used as a blank.

### 4.7. Evaluation of Inflammation in Plasma and Kidney

We used the antibodies for IL-β, IL-6, NFκβ, inhibitor of NFκβ (Iκβ) and TGF-β1 to assess inflammation. The protein concentrations in plasma or kidney cortex were determined using the Bradford method. Equal protein concentrations (7.5 µg) were denatured in a gel loading buffer at 85 °C for 5 min, then loaded onto 10% SDS-polyacrylamide gels, transferred to polyvinylidene difluoride (PVDF) membranes and incubated at 4 °C overnight with primary antibody (dilution 1:1000) diluted in phosphate buffered saline with Tween 20. The protein bands were visualized with enhanced chemiluminescence reagents (Clarity Western ECL Substrate, Bio-Rad, Hercules, CA, USA) and analyzed, and their intensity was quantified using a Kodak Electrophoresis Documentation and Analysis System 290 (EDAS 290).

### 4.8. Statistical analyses

All values are expressed as mean ± standard error of the mean (SEM). Statistical significance was determined by one-way ANOVA followed by Bonferroni analysis using Prism 5 (GraphPad, San Diego, CA, USA) software. Differences were considered significant when *p* < 0.05.

## 5. Conclusions

Our results have shown that allicin effectively decreased the oxidative stress and the systemic and renal inflammation in the experimental model of diabetes. Additionally, we observed anti-inflammatory effects from allicin, presumably mediated by their effects on NFκβ. Our study has potential clinical application because the therapy is aimed primarily at blocking mechanisms and delaying the progression of diabetic nephropathy to provide better quality of life to the patient.
